# Effects of Social Interactions and Foundational Training on Behavior, Temperament, and Hormone Levels in Weanling Horses

**DOI:** 10.3390/ani16010142

**Published:** 2026-01-04

**Authors:** Yeonju Choi, Youngwook Jung, Carissa L. Wickens, Minjung Yoon

**Affiliations:** 1 Equi-path Healing & Leadership, Yesan-gun 32445, Republic of Korea; yjchoi2031@gmail.com; 2 Department of Horse, Companion and Wild Animal Science, Kyungpook National University, Sangju 37224, Republic of Korea; 3 Department of Animal Science and Biotechnology, Kyungpook National University, Sangju 37224, Republic of Korea; wook02070@gmail.com; 4 Department of Animal Sciences, University of Florida, Gainesville, FL 32611, USA; cwickens@ufl.edu; 5 Research Institute for Innovative Animal Science, Kyungpook National University, Sangju 37224, Republic of Korea

**Keywords:** horses, social behaviors, training, hormones, human–animal interactions

## Abstract

Horses are social animals, with early life experiences playing a crucial role in their physiological and behavioral development. This study explored the influence of herd dynamics and foundational training on behavioral and hormonal changes in weaned foals. We examined 13 six-month-old Quarter Horse foals over a three-month training period, performing behavioral observations, temperament assessments, and hormone analyses at the start, midpoint, and end of the training. The results indicated that affiliative behaviors decreased significantly, while agonistic behaviors first increased and then decreased. Cortisol levels consistently declined throughout this study, whereas oxytocin levels remained stable. Linear regression analysis showed that cortisol was positively associated with affiliative behaviors and influenced by fearfulness and stubbornness. Conversely, oxytocin was positively associated with friendliness. The handlers’ proficiency significantly affected the foals’ outcomes; those managed by more skilled handlers exhibited significant improvements in confidence, friendliness, and reductions in cortisol, fearfulness, and stubbornness. These findings suggest the importance of handler skill in influencing equine behavior and hormonal balance. In conclusion, this study demonstrated the significant impacts of socialization and training on the behavioral, temperamental, and hormonal profiles of weaned foals, confirming the importance of handler expertise in fostering desirable traits in young horses.

## Simple Summary

Young horses undergo important physiological and behavioral changes during early development, and their social environment and training influence these processes. In this study, we monitored weaned foals for three months to examine how herd interactions and basic training affected their behavior, temperament, and hormone levels. We found that affiliative behaviors decreased, whereas agonistic behaviors initially increased and subsequently decreased. Cortisol levels steadily declined, indicating adaptation to the environment, while oxytocin levels remained stable. Cortisol was associated with affiliative behavior and with temperament traits including fearfulness and stubbornness, while oxytocin was linked to affiliative behavior and the temperament trait of friendliness. Importantly, the proficiency of the handler influenced fearfulness, stubbornness, confidence, friendliness, and cortisol levels. These findings highlight the importance of early social experiences and skilled handling in supporting the positive development of young horses.

## 1. Introduction

Early life experiences are crucial for the psychological and behavioral development of young horses, significantly affecting their behavior, learning, and adaptation to various environments and situations throughout their lives [1]. The development of behaviors and personality in young horses is influenced by genetics, individual experiences, and their environment [2,3,4]. Understanding this developmental process is not only important for improving our knowledge of equine cognition and welfare but also plays a significant role in enhancing training and management practices.

Early contact with humans is also an important component of foal development. Positive handling experiences during early life have been shown to reduce fearfulness, improve manageability, and facilitate habituation to routine procedures [5,6]. Such experiences contribute to emotional regulation and support the development of a more cooperative temperament later in life. Similarly, foundational or early training establishes basic communication between foals and humans, promotes clearer behavioral responses, and can reduce stress associated with unfamiliar handling. Given that the post-weaning period represents a sensitive developmental phase characterized by social and environmental challenges, appropriate human interaction and structured training may play a crucial role in supporting behavioral stability, welfare, and learning capacity in young horses.

Social experiences play a major role in developing social skills and hierarchies, essential for survival and integration into social herds. Previous research has emphasized the importance of early social experiences in shaping the behaviors and stress responses in young horses [7,8]. Specifically, social interactions are fundamental components, functioning in the formation of social bonds, establishment of hierarchies, and overall group cohesion. These behaviors reflect emotional states and are influenced by various internal and external factors.

Temperament, defined as the innate predisposition towards certain patterns of behavioral responses, significantly shapes the interaction of foals with their environment, handlers, and peers [9,10]. It influences the adaptability of horses to training and management practices [11]. Understanding the development and modulation of these temperament traits is important for the implementation of training programs, thereby enhancing training efficacy and animal welfare.

Hormones are chemical messengers produced and released by specific endocrine glands, playing a crucial role in regulating animal behavior and emotions [12]. Studies investigating the relationship between behavior and hormones have identified hormones such as cortisol and oxytocin as potential biomarker for monitoring behavior and temperament to assess well-being in mammals [13,14]. These hormones not only influence behavior but are also affected by behavior and environmental factors through a feedback loop [15].

Cortisol, commonly known as the “stress hormone”, is critical in the body’s response to stress. It is produced by the adrenal glands, and its level increases during stressful situations. The hypothalamus signals the pituitary gland to release adrenocorticotropic hormone, which in turn induces the release of cortisol, making it a common stress marker for both domestic [16] and companion animals [17,18]. In horses, salivary cortisol levels are significantly related to stress and performance [19,20].

Oxytocin plays a significant role in social bonding and stress regulation [21]. Produced in the hypothalamus and released by the posterior pituitary gland, it affects both brain and body. Various social signals can trigger the release of oxytocin, promoting social bonding [22], affiliation [23], and human–animal interactions [13]. Studies have shown that oxytocin levels are closely related to positive emotions in horses [24,25], suggesting its role in establishing positive human-horse interaction.

This study aimed to investigate the effects of social interactions and training on behavioral, temperamental, and hormonal changes in weaned horses. We hypothesized that positive social experiences and interactions with humans would correlate with behavioral adaptations and alterations in hormone levels. To test this hypothesis, we employed behavioral and temperament observations and hormone analysis as horses established social relationships with each other and underwent a training program after weaning.

## 2. Materials and Methods

### 2.1. Animals

This study involved 13 Quarter horse foals (7 fillies and 6 colts), with an average age of 177 ± 27 days, born between January and April 2022 at the Horse Research Center, University of Florida. Prior to weaning, mares and their foals were maintained in two pasture-based herd groups, allowing the foals to develop regular social contact with familiar peers. During the weaning process, individual mares were gradually removed while the foals remained together, helping to minimize social disruption and maintain familiarity among foals. After weaning, the foals continued to be housed together in their post-weaning social groups until their relocation to the Horse Teaching Unit (HTU) of the University of Florida to participate in a training program. These groups included both fillies and colts. Before relocation, the foals were not trained or handled. Routine caregiving and daily management were performed collectively by experienced staff members of the HTU, with procedures applied uniformly across all foals. While at the HTU, they received ad libitum access to Italian ryegrass hay and water, supplemented with 1.5% of their body weight in concentrates twice per day.

### 2.2. Training

**Figure 1:**
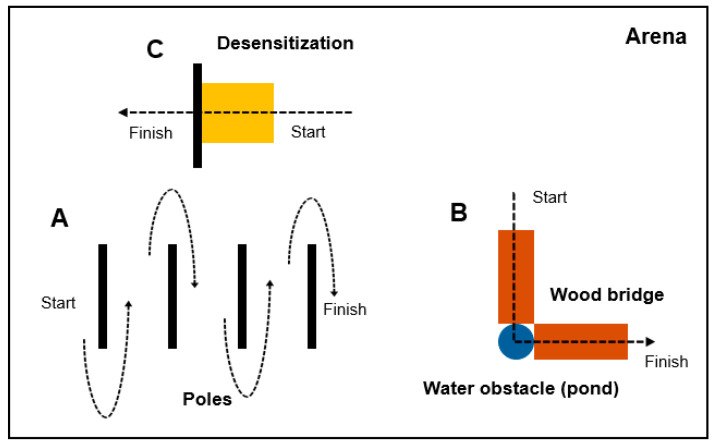
Diagram for horse training arena. Three types of obstacles were used: poles arranged in a line (**A**), a combination of wood bridges and water pond (**B**), and a yellow tarp for desensitization (**C**). Foals first trotted a serpentine path around the poles led by handlers, then walked across the wood bridges and water pond, and finally were introduced to the yellow tarp to observe their responses.

This study was conducted at the Horse Teaching Unit of the University of Florida from September to December 2022. During this period, the foals participated in a training program that included fundamental exercises such as haltering, leading, and desensitizing in an outdoor arena equipped with several obstacles (Figure 1). Each foal was paired with a specific handler, a student from the University of Florida, and this pairing remained constant throughout the program. Training sessions lasted two hours per day, twice a week. Outside of these sessions, the foals were allowed to interact and roam freely.

All handlers received instruction from faculty prior to the start of this study and followed a standardized interaction protocol throughout the training period. All student handlers were consistently supervised and guided by two primary instructors with extensive experience in young horse handling and training, ensuring uniform oversight and consistency across all handler–foal pairs. Training was based primarily on negative reinforcement using pressure-release techniques, with handlers applying light pressure followed by immediate release upon the desired response. Each session began with grooming and calm physical contact to familiarize handlers with their assigned foals before moving the training arena. A demonstration of the daily training objectives was conducted by an experienced instructor before handlers practiced the exercises with their foals, helping to maintain consistency in the interaction style and training approach among participants. Daily training routines and exercise sequences were kept as consistent as possible across all foals to minimize variability in handling and training exposure. Although minor individual differences in handler communication could not fully controlled, aversive techniques, physical punishment, or excessive pressure were strictly prohibited to ensure welfare and standardization across sessions.

### 2.3. Behavior and Temperament Assessment

Behavior observations and temperament assessments were conducted at three points during the training program: the beginning, middle, and the end. Social behaviors, including affiliative and agonistic interactions (Table 1), were observed in the pasture where foals freely interacted within the herd. Observations were performed twelve times in total, conducted twice daily, from 9:00 to 11:00 and from 13:00 to 15:00, over two consecutive non-training days at each time point. Behavioral counts from morning and afternoon sessions were summed to generate a daily value, and the two daily values were averaged to produce the final frequency used for analysis.

Temperament assessments were performed twice at each observation point by the same trained observer while handler worked with the foals. Temperament traits, such as calmness, excitability, fearfulness, confidence, stubbornness, and friendliness, were evaluated using a 10-point scale (Table 2). Prior to data collection, observers reviewed scoring criteria and conducted practice scoring to ensure consistency. At the end of the training program, the adeptness of handlers was evaluated by three faculty judges using a structured rubric, with all evaluators being faculty or University staff members who have many years of experience with horses and are involved in undergraduate teaching. The individual scores assigned by the three judges were averaged to produce a composite adeptness score for each handler. Handlers were then divided into two groups based on a median split of the composite score for statistical analysis. This post hoc grouping was conducted solely for analytical purposes and did not affect the training itself. Weather conditions remained stable throughout all observation periods, with no precipitation, strong winds, or extreme temperatures. Therefore, environmental variation was unlikely to influence the data.

### 2.4. Saliva Collection

Saliva collection was performed at three points during the training program: at the beginning, mid-term, and end. All collections occurred between 9:00 and 11:00 a.m. to account for the circadian rhythm of hormones. The synthetic swabs (Salivette^®^, Sarstedt, Nümbrecht, Germany) were used, which is specifically designed for collecting saliva. These swabs have been validated for their efficacy in measuring salivary hormones across various animal species [24,26,27,28,29]. Prior to the collection, foals were acclimated to the saliva collection procedure to mitigate stress-induced variations in hormonal measurements. For the collection, the swabs were attached to a soft rope and gently introduced into the mouths of foals for a duration of 1–2 min. Upon saturation, the swabs were immediately transferred to polypropylene tubes and subjected to centrifugation at 1000× *g* for 2 min to separate the saliva. The samples were stored in −80 °C until used for analysis.

### 2.5. Hormone Assay

The concentrations of cortisol and oxytocin were quantified using commercially available Enzyme-Linked Immunosorbent Assay (ELISA) kits, following the manufacturer’s protocols, and conducting analyses in duplicate. Cortisol ELISA kit (ADI-900-071, Enzo Life Sciences, Farmingdale, NY, USA) was used for cortisol analysis. The sensitivity was 56.72 ng/mL, and the average intra- and inter-assay coefficient of variation was 10.5% and 13.4%, respectively. Oxytocin concentrations were measured using oxytocin ELISA kit (MBS2700454, Mybiosource, San Diego, CA, USA), with a sensitivity of 4.9 pg/mL and intra- and inter-assay coefficients of variation of 5.8% and 18.4%. Cortisol concentrations were calculated using a 4-parametric logistic curve fitting, while oxytocin concentration was calculated through regression analysis in line with the recommended protocol.

### 2.6. Statistical Analysis

Statistical analysis was performed using R Studio (version 4.3.3). The normality of variables was assessed using the Shapiro–Wilk test. A mixed model for repeated measurements was utilized to compare frequencies of affiliative and agonistic behaviors, as well as each temperament trait, cortisol, and oxytocin concentration across different time points. Post hoc Tukey tests were employed to identify specific differences. The effects of handler adeptness on temperament traits were examined also using a mixed model for repeated measurements with period effect. To analyze the relationship between behaviors, temperament traits, and hormone concentrations, a linear regression model was employed. Due to a strong negative correlation between calmness and excitability (*r* = −0.969) in temperament traits, excitability was excluded to prevent multicollinearity in the analysis of temperament traits with behaviors and hormone levels. A *p*-value less than 0.05 was considered statistically significant.

## 3. Results

### 3.1. Behavior and Hormone Level Changes in Foals

**Figure 2:**
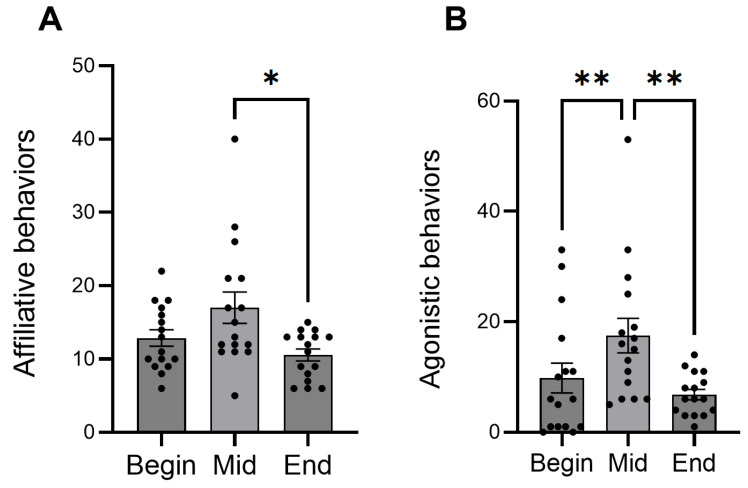
Mean numbers of the frequency of affiliative (**A**) and agonistic behaviors (**B**) showed by foals at different time points. Dots indicate individual data points. Asterisks indicate statistically significant differences (* <0.05, ** <0.01).

**Figure 3:**
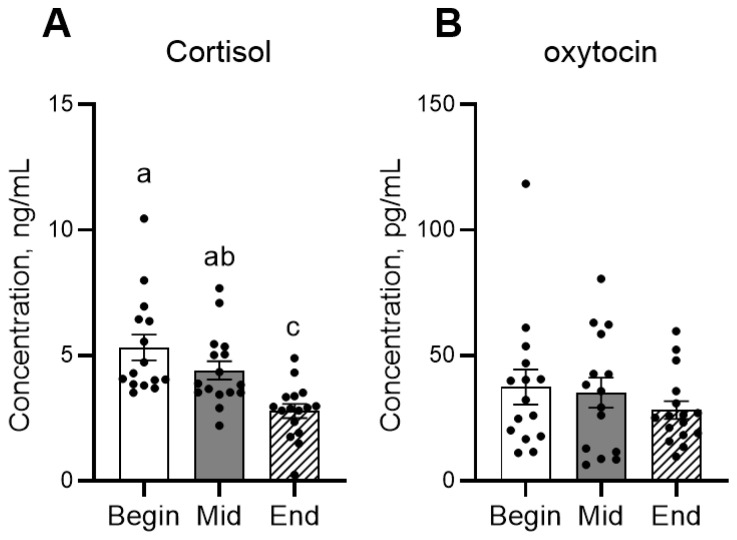
Mean concentrations of cortisol (**A**) and oxytocin (**B**) in foals across different time points. Dots indicate individual data points. Different letters indicate statistically significant differences.

In the behavior observation, the frequency of affiliative behavior significantly decreased from mid-term to the end of the period (Figure 2A). Agonistic behaviors significantly increased from the beginning to mid-term, then decreased at the end (Figure 2B). In the hormone analysis, concentrations of cortisol significantly decreased during the experimental period, whereas oxytocin remained constant (Figure 3).

### 3.2. The Relationships of Hormones, Behaviors, and Temperament Traits

In examining the association between hormone levels and interactive behaviors in horses, the linear regression analysis revealed that cortisol levels were positively influenced by affiliative behaviors, whereas agonistic behaviors had no significant effect (Table 3). Oxytocin levels were also affected by affiliative behaviors, but not by agonistic behaviors (Table 3). The analysis of hormone levels and temperament traits revealed that cortisol levels were positively influenced by fearfulness and stubbornness, whereas oxytocin levels were positively affected by friendliness (Table 4).

### 3.3. Adeptness of Handlers, Hormone Levels, and Temperament Traits

**Figure 4:**
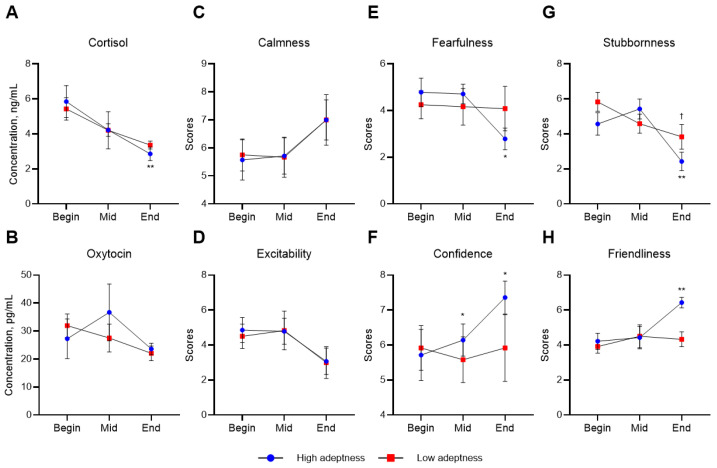
Mean concentrations of cortisol (**A**) and oxytocin (**B**), and mean scores of calmness (**C**), excitability (**D**), fearfulness (**E**), confidence (**F**), stubbornness (**G**), and friendliness (**H**) of foals in high or low adeptness groups. Asterisks indicate a significant difference in high adeptness group, compared with the beginning (* <0.05, ** <0.001). Daggers indicate significant difference in low adeptness group, compared with the beginning († <0.05).

The adeptness of handlers influenced changes in hormone levels and temperament in horses (Figure 4). Horses handled by more skilled individuals showed significantly decreased cortisol levels at the end of this study compared to the beginning. Additionally, their fearfulness and stubbornness decreased significantly, while their confidence and friendliness increased.

## 4. Discussion

The present study observed changes in behavior, temperament, and hormone levels of foals during the study period. It demonstrated that group socialization and foundational training significantly influence the development of weaned horses. The reduction in affiliative behaviors throughout the period may indicate an adjustment to the environment and a decrease in the need for social reassurance, as foals become more independent and confident in their surroundings. Although the initial increase in affiliative behaviors was not statistically significant, it suggests a potential initial response to instability of hierarchy or unfamiliarity with the environment, supported by the results of previous research [30]. The significant change in agonistic behaviors, with an initial increase followed by a decrease, could reflect the establishment of social hierarchies and the adaptation to the relationships as foals showed decreased affiliative interactions. This dynamic is commonly observed in equine social interaction as horses adapt to their environment and the herd hierarchy stabilized [31].

The temperament assessments showed the emotional and psychological development of the foals over the study period. The observed significant increase in confidence and friendliness, with decreases in fearfulness and stubbornness, indicates the positive impact of consistent foundational training on promoting a more balanced and manageable temperament in young horses. This findings aligns with previous study [24] which revealed early foundation training could encourage young horses to exhibit more positive behaviors during exercise. The stability of confidence, scores throughout the period suggests this trait may be more innate and less influenced by short-term training. Alternatively, it could indicate that the training provided was not sufficient to elicit changes in these specific temperaments.

Hormonally, significant decreases were observed in cortisol levels throughout this study. Cortisol is well known for its role in coping with stress [21,32]. The observed decrease in cortisol levels suggests a reduction in stress levels and a decrease in dependence on social reassurance, rather than reduction in social bonding itself. This hormonal response supports the behavioral observations suggesting an overall positive adaptation during the post-weaning period. Fluctuation in herd dynamics can induce increased stress levels and physiological alterations [33]. Foals were separated from their dams and moved to a new environment at the beginning of the study period, which can be a critical stressor. Elevated stress levels during the weanling process are well documented in previous studies [34,35]. The lack of significant differences in cortisol and oxytocin levels suggests that physiological response may not always manifest through behavior itself. This finding is aligned with this study, which showed a gap between behaviors and hormone levels in stress-induced conditions [25].

To further interpret the decline in cortisol concentrations observed during this study, it is helpful to consider the physiological mechanisms that underlie stress adaptation after weaning. From a neuroendocrine perspective, the decrease in salivary cortisol observed here likely reflects an adaptation of the hypothalamic–pituitary–adrenal axis to repeated social and environmental challenges. During emotional or physical stress, corticotropin-releasing hormone from the hypothalamus stimulates adrenocorticotropic hormone release from the pituitary, ultimately increasing cortisol secretion from the adrenal cortex [36]. With progressive habituation to the new environment and training context, reduced activation of this circuitry may underline the declining cortisol concentrations observed in the foals, consistent with the behavioral evidence of improved adjustment.

The results showed that foals with higher cortisol levels tended to be more fearful and stubborn. Cortisol is known for stress hormone, which elevate in response to physiological and psychological stress [37]. Fear is a functional emotion necessary for rapid evasive action when animals encounter danger. This immediate reaction is driven by the activation of the adrenomedullary system, and corticosteroids are released in response to stress [38]. The increase in cortisol levels in foals exhibiting fearfulness aligns with previous studies [39,40], indicating fearful animals perceive more situations as threatening, which triggers the stress response. Stress can also influence conflict behaviors in horses during handling [41,42]. Thus, the association of higher cortisol levels with stubbornness may be interpreted as a response to chronic stress conditions, possibly arising from continual resistance against handling or training.

In this study, foals with higher oxytocin levels were more friendly toward handlers during training sessions. Oxytocin is synthesized in the hypothalamus and released both peripherally and centrally, where it modulates social bonding, affiliative motivation, and positive social approach behaviors [21,22]. These observations suggest that higher oxytocin levels may facilitate greater social engagement and more positive interactions in foals. The role of oxytocin in social interactions has been documented in various species, indicating a link to prosocial behaviors and increased positive interactions [43,44,45]. In equine studies, oxytocin has been associated with positive emotional states [25] and increased friendliness in horses [46]. Additionally, research on young horses observed that horses showing more affiliative contact and fewer discomfort behaviors had increased levels of salivary oxytocin during foundational training [24]. Therefore, our findings and previous studies suggest that oxytocin may play a pivotal role in the development of young horses by enhancing positive human-horse interactions.

In examining the influence of handler adeptness, a clearer pattern was showed in cortisol responses than in oxytocin. Foals handled by individuals with higher adeptness exhibited a significant reduction in salivary cortisol concentrations by the end of the training period, whereas those managed by less skilled handlers did not demonstrate hormonal change across the same timeframe. Cortisol is widely recognized as a physiological indicator of internal and external stress responses [47], and weaning is known to impose considerable psychological and environmental challenges due to separation from the dam, introduction to unfamiliar companies, and exposure to novel stimuli [35]. Foundational training during this period, conducted in this study, primarily focuses on desensitization to environmental and social novelty, and the consistency and clarity of cues provided by experienced handlers may facilitate more effective habituation. This interpretation aligns with the observed decrease in fearfulness and stubbornness in the high-adeptness group, both of which are behavioral response commonly associated with uncertainty, discomfort, or resistance in unfamiliar handling contexts [48]. As foals become progressively familiar with new training demands through successful desensitization, the reduction in these temperament traits would be expected to accompany lower cortisol secretion.

Conversely, oxytocin concentrations did not differ significantly over time regardless of handler adeptness, although friendliness scores increased only in the high-adeptness group. While previous studies demonstrated that affiliative human-horse interactions can increase oxytocin release immediately following positive physical contact or bonding activities [24,26], such effects have typically been observed when samples were collected directly after intimate interaction events such as grooming, stroking, or close physical engagement. In contrast, saliva sampling in the present study was conducted at times unrelated to training sessions or direct contact with handlers, and therefore may not have captured short-lived oxytocin responses triggered by acute affiliative contexts. This methodological difference may explain why behavioral improvements in friendliness did not correspond to measurable hormonal changes.

The divergence between cortisol and oxytocin patterns may reflect fundamental differences in their physiological roles and temporal dynamics. While cortisol responds robustly to cumulative stress adaptation and therefore aligned closely with reductions in fearfulness and stubbornness in the high-adeptness group, oxytocin secretion is known to be highly context-dependent and driven by acute affiliative events rather than gradual habituation processes. Because saliva sampling in the present study was performed independently of direct social interaction with handlers, transient increases following bonding-type contact may not have been captured. Furthermore, baseline oxytocin changes may require stronger or more prolonged affiliative relationships than those provided by a three-month foundational training program focused primarily on task-oriented handling rather than sustained emotional bonding. Thus, the stable oxytocin levels observed do not necessarily indicate an absence of social connection, but rather reflect methodological constraints and the distinct neurobiological pathways underlying stress adaptation and social bonding.

Our findings showed that foals handled by more proficient handlers had lower cortisol levels and more favorable temperament traits, suggesting that the quality of handling may influence how young horses adapt to early training. Experienced handlers tend to deliver clearer and more consistent cues, apply reinforcement with more accurate timing, and recognize early signs of tension or discomfort more readily. Such skills reduce ambiguity during training and may help minimize unnecessary activation of the HPA axis, thereby supporting calmer and more confident behavioral responses in foals. In addition, research examining equestrian experience suggests that knowledge and expertise can influence training decisions and approaches, even though direct behavioral differences between experience levels are not always clearly demonstrated [49,50]. Together, these perspectives provide a theoretical framework for understanding the associations observed in our study.

A limitation of this study is the absence of pre-weaning baseline cortisol and oxytocin data, which restricts our ability to quantify the absolute magnitude of hormonal change directly attributable to the weaning transition. Although the first sampling point represented the initial physiological state immediately following weaning and relocation, it may not fully reflect the pre-weaning hormonal baseline under calm conditions. Therefore, absolute interpretation of hormonal changes should be made cautiously, and future studies incorporating pre-weaning sampling would offer more precise insight into the pattern of endocrine adaptation.

## 5. Conclusions

In conclusion, this study observed significant effects of temporal group socialization and training on the behavioral, hormonal, and temperamental changes in weaned foals. It also demonstrated the relationship between social interactions within the herd, between humans and horses, and hormone levels. These findings could contribute valuable knowledge to improving management practices that support the welfare and development of young horses.

## Figures and Tables

**Table 1:** Ethogram used to assess affiliative or agonistic interactions of horses.

Types	Behavior	Description
Affiliative	Mutual groom	Members of a herd positioned adjacent to one another, typically aligning head-to-shoulder or head-to-tail, engage in reciprocal grooming through gentle nipping, nuzzling, or rubbing actions
	Huddle	Two or more companions within a herd positioned closely together, often adopting a similar stance or posture without displaying any aggressive behaviors
	Follow	To move along the path of another horse, typically matching its gait without attempting to direct the movement, challenge, or overtake the leading horse
	Approach	A horse approaching another horse, halting within reaching distance, and standing still for at least 5 s without displaying any aggressive behavior
	Olfactory investigation	Exploratory smelling, involving sniffing different areas of another’s head and/or body, often beginning with mutual sniffing while facing each other
	Snapping	Chewing with the lower jaw moving up and down, usually with an open mouth and exposed incisors. Typically, the head and neck are extended, and the ears are relaxed and positioned back or to the side
	Head on neck, back, or rump	Resting the chin or entire head on the upper surface of the neck, body, or rump of another horse
Agonistic	Strike	Swiftly extending one or both front limbs, aiming towards another horse with the intention of making contact
	Strike threat	Quick extension of one or both front limbs towards another horse, stopping just short of making contact
	Bite	Stretching the neck towards another horse, with jaws opening and closing rapidly as if attempting to grasp the flesh
	Bite threat	Mimicking a bite motion with an open mouth and exposed teeth, and extending the neck towards another horse, but without actually making contact
	Kick	Lifting one or both hind legs off the ground and thrusting them backward towards another horse, while the forelegs support the body’s weight
	Kick threat	Similar to a kick, but with hind legs lifted slightly off the ground and tensed, ready to strike, without actually making contact
	Nip	Resembling a bite, but with the mouth less widely opened and teeth closing on only a small area of flesh
	Rear	Elevating the forequarters high into the air while the hind legs remained grounded, creating a nearly vertical stance
	Chase	One horse chasing another, often at a gallop, displaying aggressive behavior such as pinning the ears, baring teeth, and attempting to bite the pursued horse’s rump and tail
	Displace	Horse advancing toward another and occupying the exact spot the other horse vacated
	Push	Applying pressure with the head, neck, shoulder, chest, body, or rump to displace or pin the target horse against an object
	Ear pinned back	Flattening or positioning the ears behind vertical or flat against the head
	Avoidance	Engaging in movements to maintain or increase distance from an approaching horse

**Table 2:** The parameters used for evaluation of the temperament of the horse and related score.

Temperaments	Description	Scale (Not at All ↔ Very Much So)
Calmness	Remains composed of new or startling situations	1 ↔ 10
Excitability	Maintains concentration without being distracted by the environment	1 ↔ 10
Fearfulness	Tendency to get scared easily	1 ↔ 10
Confidence	Manifests as calm assurance in handling new or challenging situations	1 ↔ 10
Stubbornness	Shows resistance or reluctance to follow commands	1 ↔ 10
Friendliness	Friendly and approachable and interacts with others in a friendly manner	1 ↔ 10

**Table 3:** Linear regression results between hormone levels and interactive behaviors.

DV	IV	Coefficients	Standard Error	*t*-Value	*p*-Value	VIF
Cortisol	(Intercept)	0.045	0.234	0.193	0.847	
	Affiliative	0.517	0.203	2.549	0.014	1.012
	Agonistic	0.002	0.026	0.089	0.930	1.012
	R^2^ = 0.140, adjusted R^2^ = 0.097 *F* = 3.280, *p* = 0.048, Durbin–Watson = 1.895					
Oxytocin	(Intercept)	1.953	0.252	7.734	<0.0001	
	Affiliative	0.501	0.231	2.168	0.035	1.044
	Agonistic	0.039	0.088	0.442	0.660	1.044
	R^2^ = 0.039, adjusted R^2^ = −0.018					
	*F* = 0.678, *p* = 0.514 Durbin–Watson = 2.494					

DV = dependent variable; IV = independent variable; VIF = variance inflation factor.

**Table 4:** Linear regression results of hormone levels and temperament traits.

DV	IV	Coefficients	Standard Error	*t*-Value	*p*-Value	VIF
Cortisol	(Intercept)	−4.567	3.793	−1.204	0.237	
	Calmness	−0.129	0.226	−0.571	0.571	2.986
	Fearfulness	0.812	0.320	2.561	0.015	6.260
	Confidence	0.570	0.314	1.811	0.079	5.832
	Stubbornness	0.391	0.151	2.582	0.014	1.160
	Friendliness	0.236	0.288	0.819	0.418	2.325
	R^2^ = 0.357, adjusted R^2^ = 0.257 *F* = 3.562, *p* = 0.011, Durbin–Watson = 1.453					
Oxytocin	(Intercept)	−28.333	26.58	−1.066	0.295	
	Calmness	1.055	1.620	0.651	0.520	3.056
	Fearfulness	3.708	2.216	1.673	0.104	5.962
	Confidence	−0.399	2.209	−0.181	0.857	5.667
	Stubbornness	2.419	1.053	2.297	0.128	1.156
	Friendliness	4.930	2.053	2.401	0.022	2.336
	R^2^ = 0.449, adjusted R^2^ = 0.357					
	*F* = 4.896, *p* = 0.002, Durbin–Watson = 1.379					

DV = dependent variable; IV = independent variable; VIF = variance inflation factor.

## Data Availability

The original contributions presented in this study are included in the article. Further inquiries can be directed to the corresponding author.
